# Preparation and Characterization of Low CTE Poly(ethersulfone) Using Lignin Nano Composites as Flexible Substrates

**DOI:** 10.3390/polym15143113

**Published:** 2023-07-21

**Authors:** Jieun Jeong, Soochan Kim, Sangsoo Yun, Xin Yang, Young Jun Kim

**Affiliations:** 1 School of Chemical Engineering, Sungkyunkwan University, Suwon 16419, Republic of Korea; juj0007@naver.com (J.J.); quizofworld@naver.com (S.Y.); 2 Department of Engineering, University of Cambridge, Cambridge CB3 0FS, UK; sk2270@cam.ac.uk; 3 Key Laboratory for Light-Weight Materials, Nanjing Tech University, Nanjing 210009, China

**Keywords:** polyethersulfone, lignin, nanocomposite film, coefficient of thermal expansion, mechanical properties

## Abstract

Polyethersulfone (PES) has outstanding thermal and dimensional stability. It is considered an engineering thermoplastic. However, its high coefficient of thermal expansion (CTE) hinders its use in automobiles, microelectronics, and flexible display areas. To overcome its high coefficient of thermal expansion (CTE), recent studies have focused on reducing its high CTE and improving its mechanical properties by adding nano-sized fillers or materials. The addition of nanofiller or nanofibrils to the PES matrix often has a positive effect on its mechanical and thermal properties, making it a flexible display substrate. To obtain ideal flexible substrates, we prepared polyethersulfone with lignin nanocomposite films to reduce CTE and improve the mechanical and thermal properties of PES by varying the relative ratio of PES in the lignin nanocomposite. In this study, lignin as a biodegradable nanofiller was found to show high thermal, oxidative, and hydrolytic stability with favorable mechanical properties. PES/lignin nanocomposite films were prepared by solution casting according to the content of lignin (0 to 5 wt.%). PES/lignin composite films were subjected to mechanical, thermo-mechanical, optical, and surface analyses. The results showed enhanced thermomechanical and optical properties of PES, with the potential benefits of lignin filler materials realized for the development of thermoplastic polymer blends.

## 1. Introduction

Flexible display has attracted tremendous attention in the portability device area because of its lightweight, foldability, and easy-to-carry and store features [1,2,3,4]. Compared to previous displays, flexible displays enable folding smartphones, tablets, curved screens, and personal wearable devices [5,6]. In response to this interest, it is necessary to develop flexible and foldable backplane substrate materials that are highly foldable, transparent, and lightweight.

An ideal flexible display needs to overcome its technological limits to be more transparent, flexible, sufficiently bendable, and rugged, according to actual foldable displays [7]. One of the critical technological goals is to develop thin flexible backplane substrates with thermal stability, and a suitable coefficient of thermal expansion (CTE) to endure high-temperature processing, transparency, and flexibility [3].

Currently, metal foil and glass materials are widely used as backplane substrates in flexible organic light-emitting diodes (OLED) displays due to their thermal stability in high-temperature processing, low CTE, and dimensional stability [3]. Nevertheless, metal foil substrates still have barriers in aspects of chemical incompatibility, rough surface, and difficulty in particle free-handling. Also, glass substrates are normally made of heavy glass with low flexibility to apply flexible displays.

To be used for flexible display substrates, they require high transparency, good thermal and mechanical stability, and a low coefficient of thermal expansion (CTE).

Recently, in place of chemical incompatibility, substrate materials such as polycarbonate (PC), polyethylene terephthalate (PET), polyethylene naphthalate (PEN), polysulfone (PSF), and polyethersulfone (PES) have been suggested as substitutes for existing metal substrates [6,8,9,10]. Flexible substrates made of the materials mentioned above have benefits in terms of chemical compatibility, lightweight, high transparency, high impact resistance, and sufficient flexibility in the “roll to roll” process [6]. Thus, these above-mentioned materials can be used to produce materials that are more rugged, thinner, and conformable.

Despite the benefits of flexible plastic materials as alternatives, there are still problems.

For example, although polyethylene terephthalate (PET) and polyethylene naphthalate (PEN) as thermoplastic semi-crystalline polymers have a suitable low CTE value below 15 ppm °C^−1^ in a temperature range from −55 to 85 °C with total light transmission (TLT) of >85% over 400–800 nm, they have insufficient thermal stability and dimensional stability. Due to their insufficient thermal stability, PET and PEN undergo undesirable physical and mechanical changes in their thermal surroundings as they have a low glass transition temperature [9,11].

Polyethersulfone (PES) is considered a promising thermoplastic material due to its high optical transparency, non-crystalline nature, and reasonable price. Moreover, it also has a high T_g_ of ~220 °C and a high optical transmission of 90%. However, PES has a very high CTE of 54 ppm °C^−1^ which can interrupt OLED-deposited processing [11].

To overcome these drawbacks, recent studies have focused on reducing its high CTE and improving its mechanical properties by adding inorganic fillers or materials. The addition of nanofiller to the PES matrix often has a positive effect on its mechanical and thermal properties.

Nhat Tri et al. investigated optically transparent Poly(ether sulfone) with a low thermal expansion coefficient by using Boehmite (AlOOH) nanowires [12]. Abdul Azeez Asif et al. reported that poly(ether sulfone) improved mechanical properties and reduced the coefficient of thermal expansion with the addition of epoxy clay ternary nanocomposites [13].

Despite these previous reports, there are still some problems with the addition of an inorganic filler due to the low compatibility between fillers and PES, which can lead to deteriorated optical transparency. In addition, an inorganic filler treatment needs extra chemical processing with organic solvents before addition to the PES matrix. These extra processing treatments with inorganic fillers often require massive amounts of organic solvent to disperse, which pollutes the environment.

Recently, renewable and biodegradable composites such as cellulose, lignin, starch, protein, etc. have attracted tremendous attention to sustain the environment [14,15]. Among biodegradable composites, one made of bio-based lignin as cellulose nanofibrils has generated tremendous interest due to its low cost of production, ecofriendly, biodegradable, and renewable properties [16,17,18]. Lignin also has a moderate price because it is an abundant material produced by the pulp and paper industry as a by-product. Lignin can reinforce the mechanical and thermal properties of a polymer matrix [19,20]. Moreover, lignin has hydrophobic characteristics and hydrolytic stability that can endure humidity conditions, which is an important characteristic in electric devices [21,22]. Also, it has the ability to absorb ultraviolet (UV) light, which can protect the device from ultraviolet (UV) light [23]. Thus, lignin-containing polymers appear to have improved thermal stability, mechanical properties, antimicrobial actions, and antioxidant properties [24].

Recently, Wang et al. demonstrated that lignin composites have benefits in terms of low CTE, mechanical properties, and high optical haze [25]. Also, Simona et al. reported that lignin-based biopolymers exhibited improved thermal and mechanical properties with low CTE [24].

Thus, in this study, we prepared PES using lignin to lower its CTE, improve its thermal properties, and enhance its mechanical properties as a flexible substrate. Using the solvent casting method, PES/lignin nano composite films were prepared according to the content of lignin (0 to 5 wt.%). The properties of PES/lignin composite films were then analyzed in terms of changes in mechanical properties, thermo-mechanical properties, transparency, and surface modification.

## 2. Materials and Methods

### 2.1. Materials

Polyethersulfone (PES) and lignin were purchased from Sigma–Aldrich (Seoul, Republic of Korea). DMF was purchased from Daejung Chemical (Seoul, Republic of Korea).

### 2.2. Preparation of Polyethersulfone/Lignin Composite Films

Polyethersulfone/lignin composite films were prepared with different lignin amounts (0.25, 0.5, 1, 2.5, and 5 wt.%) by solvent casting. The PSU-lignin mixture was dissolved in DMF and stirred vigorously for 2 h at 80 °C. The solution was dispersed using an ultrasonicator to improve the dispersion of the nanocomposite in the polymer matrix. The mixture solution was then deposited on a glass plate, and the solvent was allowed to evaporate. Films were then dried under vacuum at 80 °C overnight and at 160 °C for 1 h.

### 2.3. Characterization

To investigate the interaction between PES and lignin composite, Fourier transform infrared spectroscopy (FT-IR) spectra were recorded using a NicoletTMiSTM 50 at 500–3500 cm^−1^ with an accumulation of 64 scans.

Mechanical properties of PES-lignin films were evaluated using a universal testing machine (UTM) at a load of 250 N from a Lloyd/model 5565 and a speed of 5 mm/min^−1^ at room temperature (25 ± 5 °C). All samples were cut into 50 × 10 (mm × mm) pieces with 140 ± 10 μm thickness.

Morphology transitions of PES/lignin composite films were characterized by scanning electron microscopy (SEM) (JSM-IT800 manufacturing by JEOL, Tokyo, Japan) to confirm surface transition at a power of 5.0 kV with a platinum layer coat.

The thermal transition was characterized by DSC with AutoQ20 TA Instrument equipment (New Castile, DE, USA) using nitrogen atmospheres from 25 to 280 °C with a heating rate of 10 °C/min. To evaluate thermal stability, thermogravimetric analysis (TGA) was performed with the TA Instrument under a nitrogen atmosphere from 25 to 800 °C at a heating rate of 10 °C/min.

The CTEs of films were measured using a Seiko Extar 6000 thermal analysis (TMA) machine made by SEICO (Langenhagen, Germany). Samples were scanned from 30 to 150 °C at a ramping rate of 10 °C min^−1^. The measured CTE value was calculated with the following equation [26].

$$\alpha =\frac{∆L}{∆T}\frac{1}{L_{o}}$$

where α was the indicated coefficient of thermal expansion, $L_{o}$ was the original length, ∆L was the change in length due to thermal expansion, and ∆T was the temperature gradient. The transmittance of each PES/lignin nanocomposite film was measured with an Agilent 8543 UV-Visible spectrophotometer.

## 3. Results and Discussion

### 3.1. Characterization of PES and Lignin Composites Film

#### 3.1.1. Chemical Structure Transition

**Figure 1:**
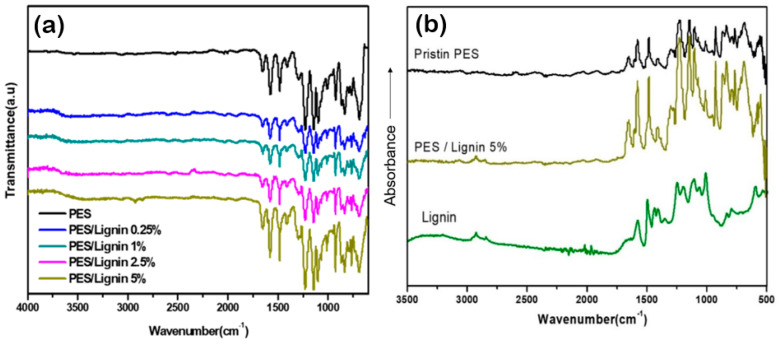
Fourier Transform Infrared spectroscopy: (**a**) Pristine PES and PES/Lignin nanocomposite blends; (**b**) Pristine PES, PES/Lignin nanocomposites 5% blends, and Lignin.

In this study, we prepared PES blended with different concentrations of lignin composites to confirm the effect of lignin. The chemical structures of neat PES and PES-lignin films were characterized by FT-IR analysis. As shown in Figure 1a, all samples showed strong peaks at 1670 cm^−1^ attributed to the stretching vibration of C=C bonds in accordance with PES structure [27,28]. Pristine PES and PES/lignin blends appeared to have peaks at 1580 cm^−1^, 1480 cm^−1^, and 1405 cm^−1^ according to the stretching vibration of integral benzene rings [28]. Two intensive peaks appearing at 1103 cm^−1^ and 1147 cm^−1^ were associated with O=S=O symmetric vibration. Also, PES and PES/lignin blends showed peaks at 1240 cm^−1^ and 1260 cm^−1^ according to the asymmetrical stretching vibration of S=O bonds [28]. Further, as low amounts of lignin nanoparticles (<5% (*w*/*w*)) were inserted into the PES matrix, there was no specific difference between PES and PES/lignin films exhibited in Figure 1.

Compared to Pristine PES, PES/Lignin blends and lignin showed different pecks at 2940 cm^−1^ and 2859 cm^−1^ corresponding to CH_2_ and CH stretching vibration, respectively, as shown in Figure 1b [29,30]. Also, PES/lignin blends and lignin showed intensive peaks at 1590 cm^−1^ and 1510 cm^−1^ attributed to C=C aromatic ring stretching vibration in the lignin [30].

#### 3.1.2. Morphology

**Figure 2:**
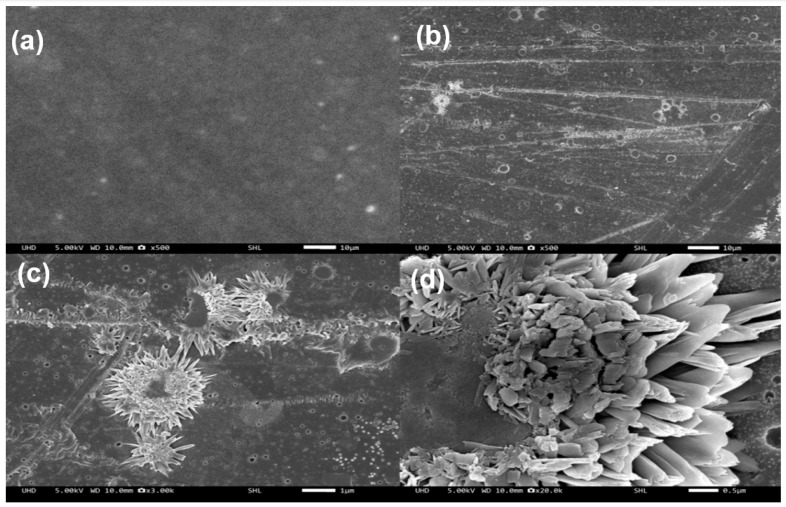
Morphology analysis of polyethersulfone and polyethersulfone(PES)/lignin nanocomposite: (**a**) Neat PES; (**b**) PES/Lignin 2.5%; (**c**) PES/Lignin 5%; and (**d**) PES/Lignin 5%.

To confirm the dispersion of lignin nanoparticles in the PES matrix, surfaces of neat PES and PES/lignin films were analyzed by SEM. Compared to the surface of PES/lignin film, the surface of neat PES film was relatively clear without any particles on the surface (Figure 2a). Compared to neat PES films, the PES/lignin film showed the presence of lignin nanocomposite in the PES matrix, which was confirmed by SEM images as shown in Figure 2b–d. 

**Figure 3:**
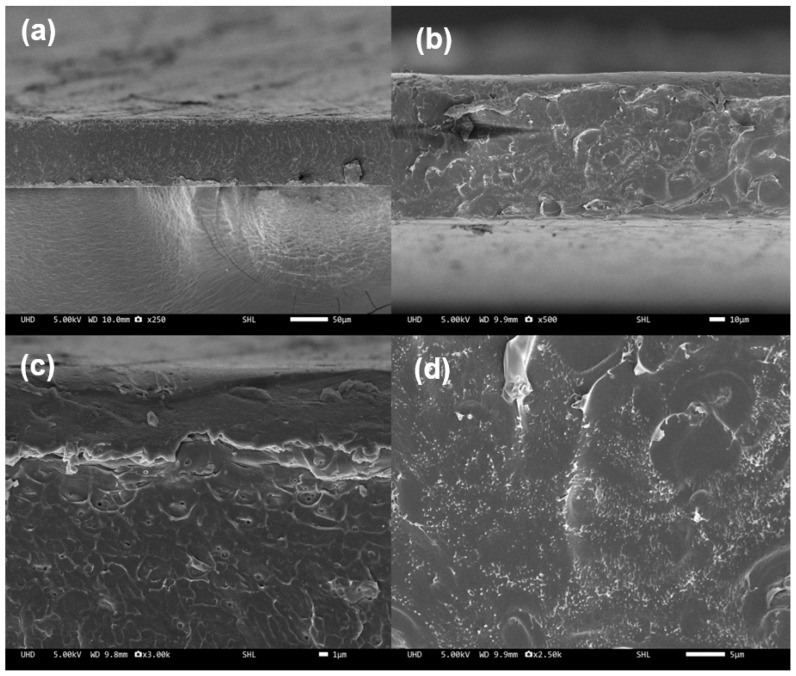
Cross-sectional image of polyethersulfone (PES) and PES/Lignin nanocomposite: (**a**,**c**) Neat PES; (**b**,**d**) PES/Lignin 2.5%..

Also, there were differences between neat PES and PES/lignin films in cross-sectional images. As shown in Figure 3, there were differences in cross-section images of neat PES and PES/lignin nanocomposite films. SEM images shown in Figure 3a,b illustrated the cross-sectional images of neat PES and lignin composite in the PES matrix. The Figure 3b cross-sectional images indicated that in the sample containing lignin nanoparticles, tortuous crack propagation, and cumulous composite were increased in the cross-sectional area. However, compared to PES-lignin composite films, neat PES films did not show any linkage or cumulous composite in the cross-sectional image, as shown in Figure 3.

### 3.2. Mechanical Properties and Thermal Mechanical Properties

#### 3.2.1. Mechanical Properties

As mechanical properties are modified by the addition of lignin nanocomposite, a universal testing machine test was conducted to confirm the transition of mechanical properties. The testing was conducted for PES film and PES blended with a very low level of lignin nanocomposite.

**Figure 4:**
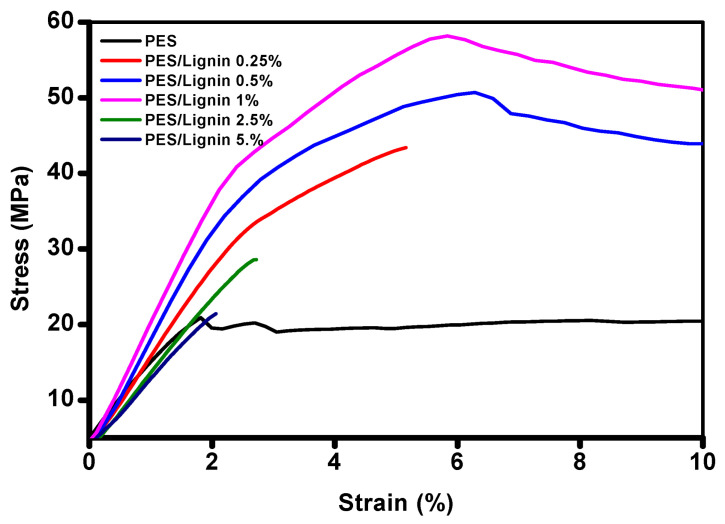
Mechanical properties of polyethersulfone and polyethersulfone/lignin nanocomposite based on their stress-strain curves.

Here, we report notable effects of nanocomposite on mechanical properties, including strength, elongation, and modulus. Results are listed in Table 1 and Figure 4. Testing films were prepared with different loading percentages of lignin composite, ranging from 0.25 to 5 wt.%.

Figure 4 shows the transition of mechanical properties in an aspect of a stress–strain plot of a neat polymer film and its blending films. As shown in Figure 4 and Table 1, the addition of a lignin composite with a very low trace can bring remarkable changes in tensile strength and modulus.

Neat PES film recorded a tensile strength of 20.9 MPa and a modulus of 1100 Mpa, as shown in Table 1. Compared to neat PES, PES-lignin films showed improved tensile strength, modulus, and strain (Table 1). Compared to neat PES, PES with only a 0.25% addition of lignin showed a tensile strength of 43.4 MPa and a strain of 5.2%, which were increased to be twice those of neat PES, while the toughness and ductility of PES-lignin films increased as the concentration of lignin content increased.

The addition of 0.5% and 1% lignin also brought incredible results, with significantly increased tensile strength (50.7 MPa and 58 MPa), modulus (1590 MPa and 1740 MPa), and strain value (6.2% and 5.8%), which were almost three times those of pure PES film. Enhancement of strength is related to lignin composites, which offer mechanical rigidity as hard segments in the PES matrix [18].

However, over 2.5% addition of lignin composite rapidly deteriorated mechanical properties. Due to the aggregation of filler within the PES-lignin matrix, PES-lignin films with high filler content became brittle, accompanied by a reduction in toughness and ductility. The addition of 2.5% or more lignin caused gradually deteriorated physical properties, showing similar mechanical properties to neat PES or worse values when the addition level was 5%.

#### 3.2.2. Thermo Mechanical Properties

As high CTE causes severe expansion of substrate at a high processing temperature (>450 °C), cracks in substrate and degradation can become major obstacles in application [25,26]. As it reflects the dimensional and thermal stability of a material at a high temperature, the CTE value is considered an important factor in the thermophysical properties of polymers used in engineering applications. In this study, to achieve a desirable low CTE for dimensional stability, we added nanocomposite in the polymer matrix to change chain mobility and developed PES with lignin nanocomposite film.

**Figure 5:**
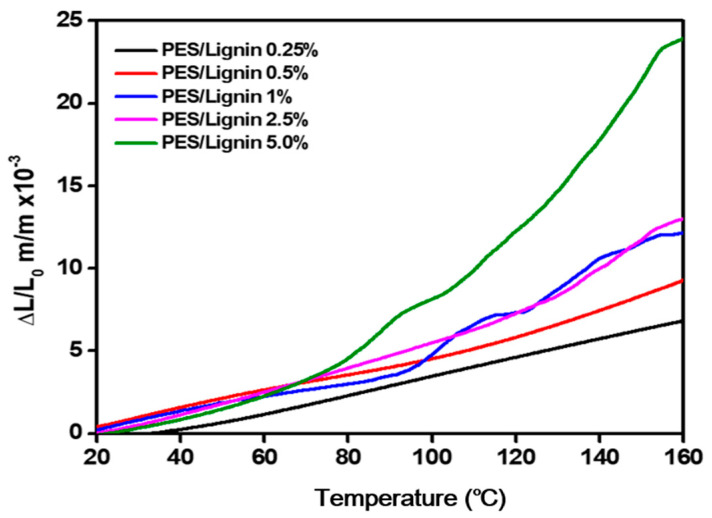
Thermo-mechanical properties of polyethersulfone and polyethersulfone/lignin nanocomposite.

The CTE value measured by TMA and its calculated result are listed in Table 1. As shown in Table 1 and Figure 5, the CTE of neat PES was 68 ppm °C^−1^ in the temperature range of 30 to 160 °C. However, the CTE value was significantly decreased with the addition of lignin nanocomposite, as shown in Table 1. The addition of a lignin composite can affect PES chemical intermolecular connections and lead to molecule motion in rigid PES molecules [31].

The CTE value gradually decreased with the addition of lignin. When the addition level was 1%, the CTE of the PES-lignin composite was 42 ppm °C^−1^, the lowest value among polymers. However, the decreasing CTE tendency of PES-lignin films started to change with the addition of over 2.5% lignin, with a phenomenon of decreasing transparency.

#### 3.2.3. Thermal Properties

**Figure 6:**
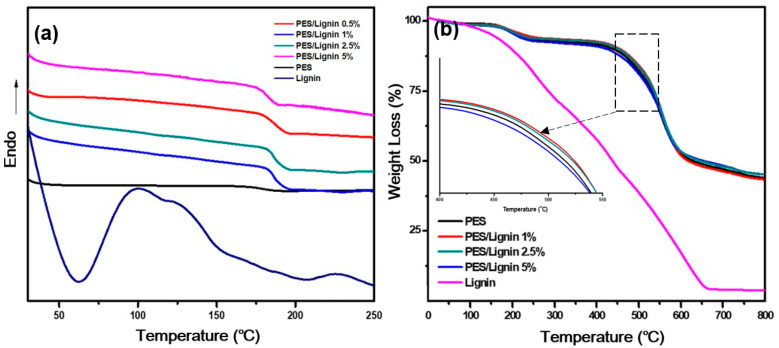
Thermo properties of polyethersulfone (PES) and PES/lignin nanocomposite: (**a**) differential scanning calorimetry (DSC) analysis of PES and PES/lignin nanocomposite; (**b**) Thermogravimetric analysis (TGA) of PES and PES/lignin nanocomposite.

Through DSC analysis, the thermal transitions of PES, PES/lignin blends, and lignin were determined. As shown in Figure 6a, the glass transition temperature (T_g_) of neat PES was 176.22 °C. After adding 0.5%, 1%, and 2.5% lignin, it increased to 185.5 °C, 186.77 °C, and 186.05 °C, respectively. Such increases were attributed to the higher presence of aromatic rings in lignin within the main chain, resulting in a decrease in the free volume fraction [32]. However, when 5% lignin was added, the T_g_ decreased to 182.15 °C due to a reduction in the polymer phase [33].

Thermogravimetric analysis (TGA) was conducted to evaluate the thermal stability of PES and PES/lignin composites. As shown in Figure 6b, all graphs exhibited two thermal drops. The first degradation occurred around 140–280 °C due to evaporation of the remaining solvent, DMF [34]. The second degradation was observed around 405–660 °C. It showed a steeper decline compared to the first degradation. This is because the thermal decomposition of the PES polymer chains can result in the formation of stable carbonaceous residues [35]. As shown in Figure 6b, PES/Lignin blends improve thermal stability compared to PES. The improvement of thermal degradation with lignin content indicated the intrinsically good thermal stability of lignin nanocomposite.

### 3.3. Optical Property

**Figure 7:**
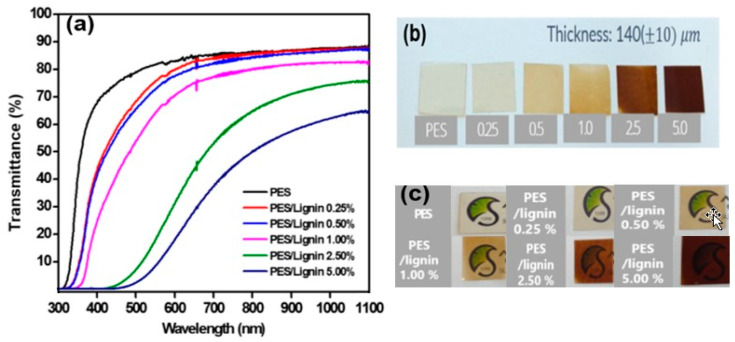
Optical properties of polyethersulfone and polyethersulfone/ligninfilms: (**a**) UV-Visible spectrophotometer analysis; (**b**) color comparison; (**c**) transparency comparison.

The transmittance of PES and PES-lignin composite films was measured with a UV-Visible spectrophotometer at a range of 300–1100 nm. Figure 7 shows the UV-vis spectra of neat PES and PES-lignin composite films. Neat PES film showed very good optical transparency in the visible light range of 400–700 nm. At 550 nm, the neat PES had a transmission of 75.8%. The PES added with 0.25% lignin composites had a transmission of 75.7%, and the PES added with 0.5% lignin composites had a transmission of 72.7%. However, the transmission result for PES added with over 1% lignin composite sharply deteriorated and became yellowish with increasing content of lignin composite, as shown in Figure 7.

## 4. Conclusions

In this study, PES/lignin nanocomposite films added with different levels of lignin, ranging from 0% to 5%, were prepared using the solvent casting method. SEM cross-sectional images confirmed that in the sample containing lignin nanoparticles, tortuous crack propagation, and cumulous composite were increased in the cross-sectional area. IR analysis revealed no significant difference in peak characteristics between neat PES and PES/lignin nanocomposite films. Compared to neat PES, films added with 0.5% and 1% lignin showed improvements in tensile strength, modulus, and strain with lower CTE values and increased Tg values, while the transmittance showed no significant difference. However, when lignin content was more than 1%, mechanical and thermal properties deteriorated, showing significant differences in transmittance compared to neat PES, although there was no significant difference in heat stability. Based on these research findings, it can be concluded that a lignin content of 1% best aligns with the objective of reducing CTE value while improving mechanical properties.

## Figures and Tables

**Table 1:** Mechanical properties of polyethersulfone/lignin nanocomposite.

Sample	Tensile Strength (MPa)	Modulus (MPa)	Strain (%)	CTE (ppm/°C)
PES	20.9	1100	1.9	68 (±3)
PES/Lignin 0.25%	43.4	1260	5.2	50 (±2)
PES/Lignin 0.5%	50.7	1590	6.2	49 (±1)
PES/Lignin 1.0%	58.1	1740	5.8	42 (±1)
PES/Lignin 2.5%	28.6	1077	2.7	70 (±2)
PES/Lignin 5.0%	21.4	980	2.1	96 (±5)

## Data Availability

Not applicable.
